# Specialties in Medicine

**Published:** 1865-12

**Authors:** 


SPECIALTIES IN MEDICINE. By HENRY D. NOYES, M.D., Surgeon to the New York Eye and Ear Infirmary. Read before the American Ophthalmological Society, June, 1865.

This is an interesting pamphlet of 16 pages, on a topic of general interest.

